# Analysis of Polymorphic Membrane Protein Expression in Cultured Cells Identifies PmpA and PmpH of *Chlamydia psittaci* as Candidate Factors in Pathogenesis and Immunity to Infection

**DOI:** 10.1371/journal.pone.0162392

**Published:** 2016-09-15

**Authors:** Sarah Van Lent, Winnok H. De Vos, Heather Huot Creasy, Patricia X. Marques, Jacques Ravel, Daisy Vanrompay, Patrik Bavoil, Ru-ching Hsia

**Affiliations:** 1 Department of Animal Production, Faculty of Bioscience Engineering, Ghent University, Ghent, Belgium; 2 Department of Veterinary Sciences, Faculty of Pharmaceutical, Biomedical and Veterinary Sciences, University of Antwerp, Antwerp, Belgium; 3 Department of Molecular Biotechnology, Faculty of Bioscience Engineering, Ghent University, Ghent, Belgium; 4 Institute for Genome Sciences and Department of Microbiology & Immunology, University of Maryland School of Medicine, Baltimore, Maryland, Unites States of America; 5 Department of Microbial Pathogenesis, University of Maryland School of Dentistry, Baltimore, Maryland, Unites States of America; 6 University of Maryland, Baltimore, Electron Microscopy Core Imaging Facility, Maryland, Unites States of America; Instituto Butantan, BRAZIL

## Abstract

The polymorphic membrane protein (Pmp) paralogous families of *Chlamydia trachomatis*, *Chlamydia pneumoniae* and *Chlamydia abortus* are putative targets for *Chlamydia* vaccine development. To determine whether this is also the case for Pmp family members of *C*. *psittaci*, we analyzed transcription levels, protein production and localization of several Pmps of *C*. *psittaci*. Pmp expression profiles were characterized using quantitative real-time PCR (RT-qPCR), immunofluorescence (IF) and immuno-electron microscopy (IEM) under normal and stress conditions. We found that PmpA was highly produced in all inclusions as early as 12 hpi in all biological replicates. In addition, PmpA and PmpH appeared to be unusually accessible to antibody as determined by both immunofluorescence and immuno-electron microscopy. Our results suggest an important role for these Pmps in the pathogenesis of *C*. *psittaci*, and make them promising candidates in vaccine development.

## Introduction

The *Chlamydiaceae* are a family of Gram-negative obligate intracellular bacteria that infect animals and humans, causing diseases with a wide range of symptoms. Among these, a significant species is *C*. *psittaci* that may cause respiratory disease in poultry and pet birds, and may also cause zoonotic psittacosis in humans. Psittacosis, or parrot fever, is usually characterized by fever chills, headache, dyspnea and cough. Chest X-rays often show an infiltrate [1]. However, the disease seems to vary considerably in severity as the clinical features of the infection can range from none to sepsis with multi-organ failure requiring admission in an intensive-care-unit [2]. People usually contract the infection via inhalation of an aerosol from droppings of infected birds. Epidemics of *C*. *psittaci* infections in turkeys are economically devastating due to high mortality rates, carcass condemnation at slaughter, reduced egg production and/or the cost of antibiotic treatment to reduce mortality and allow marketing of turkeys [3].

Little is known about the mechanisms by which *Chlamydia* species manipulate host cells and induce disease in different hosts. In spite of diverse infection strategies and symptoms, all *Chlamydia* spp. share a unique, conserved, biphasic developmental cycle. The elementary body (EB) is the infectious, metabolically dormant form of *Chlamydia*, which differentiates into the metabolically active reticulate body (RB) after internalization by the eukaryotic host cell. The developmental cycle takes place entirely inside a vacuole, called the inclusion. After several rounds of exponential growth, the RBs asynchronously differentiate into EBs. The infectious EBs are then released from infected host cell through cell lysis or inclusion extrusion, thereby closing the developmental cycle.

Chlamydial proteins are differentially produced in EBs and RBs [4,5]. Proteins present on the surface of EBs are of particular interest for vaccine development, as they are putative targets for neutralizing antibodies. Antibodies against polymorphic membrane proteins (Pmps; previously known as 90-kDa protein family; reviewed in [6]) have been detected during natural infections of humans, turkeys and sheep [7–12] and during experimental infections of specific pathogen-free turkeys [13,14]. Longbottom *et al*. [11], were first to clone and sequence four genes encoding members of the 90-kDa protein family. Genome sequencing later revealed that the *pmp* genes encode the largest membrane protein family in *Chlamydia* spp., a unique feature of the genus [15,16]. In the last decade, the Pmps have been studied intensively, particularly because the *Chlamydia trachomatis* and *Chlamydia pneumoniae* Pmp families represent a relatively high proportion of the coding capacity (3.15 to 5.1%, respectively) in the highly reduced chlamydial genome. Moreover the occurrence of the *pmp* gene family in all currently sequenced chlamydial genomes [17] suggests an important function in chlamydial biology. The observed diversity in the number of alleles, gene and protein sequences, size (90–190 kDa), and expression levels within and across *Chlamydia* spp. also suggests that Pmps may be responsible for observed differences in the pathogenesis across *Chlamydia* species.

Pmps were classified as autotransported (type V secretion) proteins, based on their N-terminal signal sequence (type II secretion), a central passenger domain and a C-terminal putative transporter domain, predicted to form a β-barrel through which the protein is secreted to the chlamydial surface [18,19]. This prediction is supported by experimental evidence for several Pmps [20–24]. The Pmps are grouped into a family based on the conserved repetitive motifs FxxN and GGA (with I, L or V at the 4^th^ position). In *C*. *trachomatis*, they have been further divided into six phylogenetically related subtypes (PmpA, B/C, D, E/F, G/I, and H) which may be able to substitute structurally and functionally for one another [17]. The passenger domain is responsible for the protein’s function [18]. Pmp6, Pmp20 and Pmp21 of *C*. *pneumoniae* (orthologs of PmpG, PmpB and PmpD of *C*. *trachomatis*, respectively) and all Pmps of *C*. *trachomatis* are proposed to function as adhesins, based on adhesion assays and specific neutralization of the infection by incubation of the host cells with the recombinant Pmps [25–27]. Up to now, anti-PmpD antibodies are the only Pmp-specific antibodies that are tested for their possible neutralizing capacity. Specific anti-PmpD and anti-Pmp21 antibodies can partially neutralize *C*. *trachomatis* and *C*. *pneumoniae* infection, respectively, *in vitro* [22,25,27]. Patients infected with *C*. *trachomatis* usually elicit high titer antibodies against a subset of the Pmps, that varies between infected individuals [28]. The different antibody profiles in patients may reflect different transcription and protein production profiles along the developmental cycle or as a result of strain variation or site specificity [21,28–32]. An attractive hypothesis is that variation of *pmp* gene expression and the resulting antigenic variation phenotype contribute to immune evasion in the infected host. Finally, Pmps were reported to be involved in host and tissue tropism [26]. Previous studies have mainly focused on the Pmps of *C*. *trachomatis* [5,23,26–28,31,33–37] and *C*. *pneumoniae* [21,22,25,38], both human pathogens, and on the zoonotic *C*. *abortus* [11,20,30,32,39,40]. However, the *pmp* gene family of *C*. *psittaci* has not been investigated so far.

We hypothesize that Pmps play an important role in *C*. *psittaci* pathogenesis and in immunity to infection. To test this hypothesis, we studied developmental expression and abundance profiles of different Pmps using quantitative real-time PCR (RT-qPCR) and immunofluorescence microscopy (IF), respectively, for *C*. *psittaci* strain Cal10. Immuno-electron microscopy (IEM) was used to assess subcellular localization of the Pmps on individual chlamydiae. As previous studies suggested a unique role in chlamydial pathogenesis for virulence genes expressed during stress, IF analyses of the Pmp products of *C*. *psittaci* were conducted under both normal and stressed conditions [31].

## Materials and Methods

### Bioinformatics analyses

We re-annotated the *pmp* genes of the *C*. *psittaci* Cal10 genome (AEZD00000000.1) using a newly developed Hidden Markov Model (HMM) as described below. Individual clustal alignments were independently run on each *pmp* subfamily within the seed set, consisting of manually curated *pmp* genes from six sequenced *C*. *psittaci* genomes (6BC, RD1, 01DC11, 02DC15, 08DC60 and C1998). Alignments were reviewed manually and it was determined that no trimming was necessary. The HMMER package (hmmbuild) was used to build a separate model for each *pmp* subfamily. All HMMs were validated using the HMMER package (hmmsearch) against a larger set of *C*. *psittaci* and *C*. *caviae* sequences.

### *Chlamydia psittaci* cell culture conditions

HeLa 229 cells (human cervical cancer cells) were seeded in 100 mm^2^ tissue culture dishes for 24h at 37°C with 5% CO_2_ in Dulbecco’s modified Eagle’s medium (DMEM, Mediatech, Herndon, VA) supplemented with 10% heat inactivated fetal bovine serum (Atlanta Biologicals, Lawrenceville, GA), gentamycin (25 μg ml^-1^; Quality Biological, Gaithersburg, MD) and fungizone (1.25 μg ml^-1^; Invitrogen, Carlsbad, CA). The medium was aspirated and cells were inoculated with *C*. *psittaci* Cal10 in SPG (0.25 M sucrose, 10 mM sodium phosphate and 5mM L-glutamic acid) at a multiplicity of infection (MOI) of 1 followed by incubation on a rocking platform for 2 h at 37°C. The unbound organisms were washed away with PBS and the bacteria were grown either under normal conditions using the above-mentioned medium, or by adding penicillin (100U ml^-1^) to the medium to induce stress-related persistence [41]. One ml SPG was added to a mock-infected dish. For all cells, addition of medium after rocking and washing marked time 0 hpi of the experiment.

### Total RNA extraction and cDNA synthesis

At 2, 6, 12, 18, 24, 32, and 48 hours post infection (hpi), total RNA was extracted from the monolayers according to the manufacturer’s instructions (TRIzol, Invitrogen). Total RNA was quantified (Nanodrop 2000, Thermo Scientific, Wilmington, DE) and the samples were treated with RNase-free amplification grade DNase I (Promega, Madison, WI) following the manufacturer’s protocol and confirmed to be DNA-free by PCR (S1 Table) for the *C*. *psittaci* Cal10 *16S rRNA* gene. One μg of total RNA was reverse transcribed (Superscript II RT, Invitrogen) with random hexamer primers (Invitrogen) following the manufacturer’s protocol. RNA and cDNA samples were stored at -80°C and -20°C, respectively.

### Primer design and validation for RT-qPCR

*Pmp*-specific regions were identified by ClustalW2 alignment. Primers for all *pmp* genes were designed using Primer3 software with the following settings: amplicon size of 100–200 bp, optimal melting temperature of 60°C and a GC content of 50–60% (S2 Table). For each primer pair, different primer concentrations (100 nM, 150 nM and 200 nM) were tested in duplicate. The concentration resulting in the best sigmoid expression curve was chosen (S2 Table). Melt curve analysis was used to ensure the specificity of the primers. RT-qPCR efficiencies for each gene were determined using slope analysis with a linear regression model. Serial dilutions of genomic DNA of purified EBs (1/5 = 8 ng μl^-1^, 1/25, 1/ 125, 1/625, 1/3125, 1/15625) were used to generate standard curves. Corresponding RT-qPCR efficiencies (E) were calculated according to the equation E = (10^(-1/slope)^-1) x 100 [42]. Ideally, efficiencies should be between 90–110%, but because of the difficulty to find specific regions in the *pmp* genes, primer pairs with efficiencies outside this range were also used (S2 Table). Obtained Cq-values were corrected for the differences in PCR-efficiencies. Primers that displayed a coefficient of correlation greater than 0.98 were selected for RT-qPCR.

## Developmental expression of *pmp* genes (RT-qPCR)

The expression of the *pmp* genes was examined by RT-qPCR on an iQ5 Real-Time PCR Detection System (Bio-Rad, Richmond, CA). Each reaction mixture contained 1 μl cDNA, the optimal primer concentration for each primer pair (S2 Table), 10 μl iQ SYBR Green Supermix (Bio-Rad) and ddH_2_O to a final volume of 20 μl. RT-qPCR reaction conditions were as follows: initial denaturation at 95°C for 3 min, 40 cycles each consisting of 30 s at 95°C and 30 s at 58°C, followed by the melting curve program (95°C for 1 min, 55°C–95°C in steps of 0.5°C each 10 s). Two biological replicates, each at the 7 different developmental times, were analyzed. Genomic DNA of *C*. *psittaci* Cal10 was used as a positive control. In addition, *tufA* encoding elongation factor EF-Tu involved in protein synthesis was also included as a positive control, because the gene is constitutively expressed throughout the developmental cycle and it is a reliable indicator of exponential growth [31]. cDNA of HeLa 229 cells, non-reverse-transcribed total RNA of *C*. *psittaci* Cal10, and ddH_2_O were used as negative controls. Data analyses were carried out with qBasePLUS software (version 2.4, Biogazelle, Ghent, Belgium) and validated reference genes (*tyrS*, *gidA*, *radA*, *map* and *16S rRNA*, not shown) were used for normalization. Developmental times were defined as early (2–6 hpi), middle (12–18 hpi) and late (≥ 24 hpi).

### RT-PCR

RT-PCR was performed on cDNA samples (24, 32 and 48 hpi, normal conditions) to evaluate the putative organization of the *pmp* genes in operons in the *C*. *psittaci* Cal10 genome. Primers spanning the intergenic regions were designed using Primer3 software (http://bioinfo.ut.ee/primer3-0.4.0/) (Table 1). *C*. *psittaci* Cal10 genomic DNA was used as a positive control while cDNA from uninfected HeLa 229 cells, non-reverse-transcribed *C*. *psittaci* Cal10 total RNA, and ddH_2_O were used as negative controls.

**Table 1:** Primers spanning the intergenic regions, used for RT-PCR to test the organization of *pmp* genes in operons.

Intergenic region	Primer	Primer sequence (5'-3')	Amplicon size (bp)	Tm (°C)	GC (%)
*pmpA*, *pmpB*	A-B-F	ACCTACTGCGAATATTCACTGTC	225	58.32	43.5
	A-B-R	GCGACGCTTTTGGTGGTATA		58.64	50
*pmpE1*, *pmpE2*	E1-E2-F	CTGAGAAATTGGGCAGGTAAAGA	197	58.66	43.5
	E1-E2-R	TCCAGATCCACATTATGCAACT		57.49	40.9
*pmpH*, *pmpG2*	H-G2-F	GCATGCCTCAACCCTATCGT	207	60.18	55
	H-G2-R	CAACGGCGCTAGATGGAAAA		58.92	50
*pmpG2*, *pmpG5*	G2-G5-F	TGCTAGACAGGATGCAACAAG	260	58.3	47.6
	G2-G5-R	GGGATCTGGGAAGCCAATTG		58.59	55
*pmpG5*, *pmpG8*	G5-G8-F	TTGTCGCCGCTACATTTTGT	561	61.58	45
	G5-G8-R	AAGACATGCTGCACGATTCG		62.35	50
*pmpG8*, *pmpG7*	G8-G7-F	ATATAGCCCCGCCGTTATCG	902	59.54	55
	G8-G7-R	GAGGGTTAGCTCCATGGACA		58.8	55
*pmpG7*, *pmpG3*	G7-G3-F	TAAATTGCCCGCCTCCTGTA	408	59.09	50
	G7-G3-R	GCTCTGTTGCAAGGATCGAG		58.99	55
*pmpG1d*, *pmpG1b*	G1d-G1b-F	TGTTGTTCCTTGAGGTGCAG	366	59.87	50
	G1d-G1b-R	TTGAGCTCCGAGGTTCTTGT		59.99	50
*pmpG1c*, *pmpG6*	G1c-G6-F	TTGATCCCCAGCTGTATTCC	419	54.4	50
	G1c-G6-R	GGTTAACCACAGCGACGAAT		55.4	50
*pmpG6*, *pmpG4*	G6-G4-F	GCTCCATTTGCATCGAGAAT	299	60.19	45
	G6-G4-R	CGTTGACATAGGAGGCAGGT		60.13	55

### Cloning of *pmp* genes

Due to the expansion of the Pmp family in *C*. *psittaci*, we decided to clone one *pmp* CDS of each Pmp subtype. Therefore, fragments of *pmpA*, *B*, *D*, *E1*, *G3* and *H* were amplified from *C*. *psittaci* Cal10 genomic DNA by PCR using Pfu polymerase (St. Leon-Roth, Germany) and primers flanked with specific restriction sites (Table 2) for subsequent cloning. The *pmpA* (aa 309–898) and *pmpH* (aa 420–942) fragments were cloned in pGEX-2T (Amersham Pharmacia Biotech, Piscataway, NJ), while *pmpB* (aa 296–955), *pmpD* (aa 321–1193), *pmpE1* (aa 313–958) and *pmpG3* (aa323-791) fragments were cloned in pET-19b (Novagen, Madison, WI). Cloned inserts were sequenced to confirm correct in-frame insertion and N-terminal fusion with the GST-tag (pGEX-2T) or His_6_-tag (pET-19b).

**Table 2:** Primers and restriction enzymes used for the cloning of the *pmp* genes.

Gene	Primer	Primer sequence (5'-3')	Vector	Restriction enzymes	Molecular mass (kDa)	
					Calculated	Apparent
*pmpA*	pmpA-F	CCCGGGTGCGGAATTTCAGGTTGAGTGC	pGEX-2T	XmaI	96	75
	pmpA-R	GGATCCAGCCAAAACTCCGCAGAAGG		BamHI		
*pmpB*	pmpB-F	GGATCCAGTGTGAAGCTCGTCTTAGC	pET-19b	BamHI	74	70
	pmpB-R	CTCGAGGCTGAATCTGGAATTGGCGG		XhoI		
*pmpD*	pmpD-F	CTCGAGCATGCGGATATCCAGTACC	pET-19b	XhoI	95	95
	pmpD-R	AAGCTTTTAAGACCACGTTCCCATATGTCC		HindIII		
*pmpE1*	pmpE1-F	AAGCTTTTAAGCATGTCGTGAGTTTGGCG	pET-19b	HindIII	74	70
	pmpE1-R	CATATGGCAGACCTTAACGGTGGAGC		NdeI		
*pmpG3*	pmpG3-F	GGATCCAGGGGAGTAGGCCTCCAGA	pET-19b	BamHI	60	60
	pmpG3-R	CATATGAACTTTTTCATTCATTCCCCTGA		NdeI		
*pmpH*	pmpH-F	GAATTCTCCATTACGAGCGATATGCGC	pGEX-2T	EcoRI	88	75
	pmpH-R	GGATCCGGGGATATGGTCTTTATCGGC		BamHI		

### Expression and purification of recombinant Pmps

*Escherichia coli* BL21 cells were transformed by electroporation and Pmp expression was induced at an OD_600_ of 0.5–0.8 upon the addition of 0.1 mM isopropyl β-D-thiogalactoside for 4 h at 28°C. GST- or His_6_-tagged protein expressing cells were centrifuged (6 000 *g* for 15 min at 4°C), resuspended in ice-cold PBS or in 50 mM sodium phosphate and 300 mM NaCl respectively. Cells were lysed by twice passing through a French Pressure cell (American Instrument Co., Urbana, IL) followed by sonication (3 x 30 s). One percent Triton X-100 (Sigma, St Louis, MO) was added and the lysates were placed on ice on a rocking platform for 30 min. Soluble and insoluble proteins were separated by centrifugation (16 000 *g* for 20 min at 4°C) and analyzed by sodium dodecyl sulfate-polyacrylamide gel electrophoresis (SDS-PAGE). Recombinant Pmps were present in the insoluble fractions, which were resuspended in buffer (50 mM Tris-HCl, 1 mM EDTA, 1 mM DTT, 8 M urea) and placed on a rocking platform (1 h, RT). Refolding of the recombinant Pmps was facilitated by overnight incubation (42°C) in the non-ionic detergent n-octyl-b-D-glucopyranoside (Biosynth, Staad, Switzerland) [43]. The samples were dialyzed three times against 1x PBS with 0.1% Triton X-100 and subsequently subjected to affinity chromatography using a Glutathione-Sepharose 4B (GE Healthcare, Little Chalfont, UK) column for GST-tagged proteins, and a TALON metal affinity resin (Clontech, Palo Alto, CA) was used for His_6_-tagged proteins according to the manufacturers’ instructions. The recovered recombinant protein fractions were subjected to SDS-PAGE and stained with Coomassie blue. Eluted fractions containing the recombinant proteins were dialyzed and concentrated with a vacuum concentrator (Spectrum Laboratories, Rancho Dominguez, CA).

### Generation and characterization of Pmp-specific polyclonal antibodies

Polyclonal antibodies (pAbs) against purified recombinant Pmp E1, H and G3 of *C*. *psittaci* Cal10 were generated by immunization of guinea pigs. Animal maintenance and experimental treatments were conducted in accordance with the ethical guidelines for animal research established and approved by the institutional Animal Care and Use Committee at University of Arkansas for Medical Sciences, more specifically this study has been approved in protocol number 2975. Two female guinea pigs (strain Hartley) were immunized with each antigen. The guinea pigs were housed individually, fed with Harlan Teklad guinea pig diet, checked twice a day and sacrificed using carbon dioxide. For each animal, 500 μg of immunogen was mixed with the same volume of Freund’s Complete Adjuvant for primary immunization and two subsequent boosters with immunogen and Freund’s Incomplete Adjuvant were administered. Previously, pAbs against recombinant PmpA, PmpB and PmpD of *C*. *caviae* had been generated in the same way (P. Bavoil, unpublished data) All pAbs were characterized by immunoblotting using purified recombinant PmpA, B, D, E1, G3 and H of *C*. *psittaci* Cal10 as well as density gradient purified EBs of *C*. *psittaci* Cal10 as antigens. The pAbs were pre-adsorbed with HeLa 229 cells and with inclusion bodies of *E*. *coli* BL21 expressing an irrelevant antigen (His_6_-tagged recombinant β-galactosidase or GST, purified by the same protocol as the recombinant Pmps) to remove any non-specific cross-reactivity. Secondary HRP-rabbit anti-guinea pig antibody (Invitrogen) was used for detection.

### Immunofluorescence microscopy

At 12, 18, 24, 32 and 48 hpi, infected HeLa 229 cell cultures (with or without penicillin) were washed with PBS and fixed for 30 min at -20°C with 100% methanol. Monolayers were blocked for 1 h with 5% fetal bovine serum and subsequently double-labeled; Chlamydiae were stained by anti-LPS-FITC (IMAGEN chlamydia test, Novo Nordisk Diagnostics, Cambridge, UK) and Pmps were observed by indirect immunofluorescence after staining with anti-PmpA, anti-PmpB, anti-PmpD or anti-PmpH primary antibodies in combination with Alexa Fluor 568-conjugated goat anti-guinea pig IgG (Invitrogen), and counterstaining with DAPI. Images were acquired and recorded manually via confocal laser scanning microscopy (Nikon A1R, Nikon Instruments Inc., Paris, France), using a 40x Plan Apo oil objective with a numerical aperture of 1.3. DAPI, FITC and AF568 were respectively excited with a 405 nm diode, a 488 nm Ar and a 561 diode laser and their fluorescence emission was respectively detected through a 440/50 nm, 525/50 nm and 595/50 nm band pass filter. The pinhole was set to 1 Airy unit and acquisition settings (laser power, gain and offset, scan speed) were kept constant throughout the experiment. Image analysis was conducted with ImageJ freeware [44] on ten independent experiments using an in-house written colocalization script for ImageJ to determine the percentage of inclusions expressing PmpA, B, D and H, as described before [45]. In brief, the analyses determined the percentage of inclusions that is positive for specific Pmp proteins (i.e. above an intensity threshold) by calculating the overlap of both binarized channels per object (inclusion). The analysis was benchmarked using a manually curated image data set with varying number of positive inclusions and varying intensity levels. Two different settings were used: setting 1 with both normal and smaller size inclusions and setting 2 with normal size inclusions only for late developmental times.

### Immuno-electron microscopy

Infected HeLa 229 cell cultures (with or without penicillin) were fixed in 4% paraformaldehyde and 0.1 M PIPES buffer (pH 7.35) at 24 and 48 hpi. Cells were removed with a cell scraper, washed, pelleted, and enrobed in 2.5% low-melting-temperature agarose. Agarose blocks were trimmed into ~1mm^3^ size, washed, dehydrated, infiltrated, and embedded in unicryl at -20°C under UV from 24 to 48 h. Ultrathin sections were cut on a Leica UC6 ultramicrotome (Leica Microsystems Inc., Bannockburn, IL) and collected onto formvar-coated Nickel grids. Immunogold labeling was performed using the guinea pig anti-PmpA, B, D and H specific pAbs followed by a secondary gold conjugated goat anti-guinea pig IgG (H&L) antibody (Electron Microscopy Sciences, Hatfield, PA). Sections were also stained using a rabbit anti-MOMP-specific serum followed by a secondary gold conjugated goat anti-rabbit IgG (H&L) antibody (Electron Microscopy Sciences). Images were acquired using a Tecnai T12 transmission electron microscope (FEI, Hillsboro, OR) at 80 keV and an AMT digital camera (Advanced Microscopy Techniques, Woburn, MA).

### Statistics

Statistical analyses were performed using R (version 3.0.3). The percentage of positive inclusions for PmpA, PmpB, PmpD and PmpH (median for PmpA, B and H and average for PmpD) at different times post-infection were compared based on 10 biological replicates by use of the non-parametric Kruskal-Wallis test (PmpA, B and H) followed by the Mann-Whitney test or the parametric one-way ANOVA followed by Tukey’s post hoc analysis (PmpD). For 24, 32 and 48hpi, the median percentage of positive inclusions for PmpA, PmpB, PmpD and PmpH, with both normal size and smaller inclusions included was compared with the median percentage of positive inclusions with only normal size inclusions, by the Wilcoxon signed rank test. The abundance of the immunogold labeling for PmpA, PmpH and MOMP was compared on 10 biological replicates by use of the Kruskal-Wallis test followed by the Mann-Whitney test. For all tests, results were considered significantly different if P < 0.05.

## Results

### The *C*. *psittaci* Cal10 genome encodes 17 predicted *pmp* coding sequences (CDSs)

**Fig 1:**
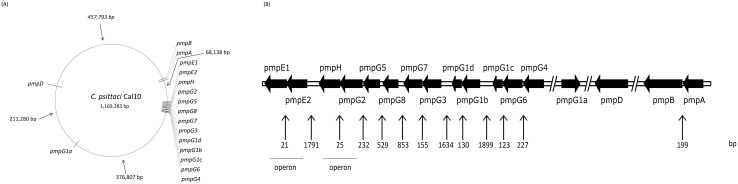
*pmp* gene organization in the *C*. *psittaci* Cal10 genome. (A) The *pmp* CDSs map to 4 distinct loci on the *C*. *psittaci* Cal10 genome. Distances (bp) between nearest loci are indicated. (B) Linear representation of the *pmp* loci. CDSs and inter-CDS regions are drawn to scale. Number of bp between 2 nearest CDSs are indicated. A break (//) is added if the inter-CDS region is bigger than 5000 bp.

We developed a hidden Markov model for the identification of the *pmp* CDSs in the *C*. *psittaci* Cal10 genome. We identified 17 *pmp* CDSs (Table 3), representing 4.1% of the genome size at 4 distant genomic loci (Fig 1A). All predicted *pmp* genes of *C*. *psittaci* Cal10, except for *pmpG1a*, are encoded on the complementary strand (Fig 1B). Eleven *pmpG* alleles are present in the genome, two of which (*pmpG1c* and *G1d*) are predicted to encode truncated products. The *pmpG8* allele codes for a 75.81 kDa protein, therewith reducing the previously determined lower molecular mass boundary (90 kDa) for Pmps [17].

**Table 3:** *pmp* genes and gene products of *C*. *psittaci* Cal10.

Pmp subtype^a^	Start position	Stop position	Size (bp)	Theoretical pI	Theoretical Mw (kDa)^b^	Locus tag
A	222944	225745	2799	8,82	101,84	G5Q_0224
B	217351	222744	5391	6,04	190,48	G5Q_0223
D	924237	928841	4602	5,22	163,72	G5Q_0827
E1	293884	296886	3000	5,86	109,95	G5Q_0290
E2	296908	299793	2883	6,60	106,57	G5Q_0291
G1a	710413	712956	2541	5,25	91,27	G5Q_0648
G1b	322009	324513	2502	5,81	89,39	G5Q_0303
G1c	326413	327735	1320	5,16	45,85	G5Q_0306
G1d	320559	321878	1317	4,96	45,34	G5Q_0302
G2	304565	307696	3129	7,55	110,88	G5Q_0294
G3	316390	318924	2532	8,01	92,34	G5Q_0300
G4	330783	333605	2820	6,24	100,51	G5Q_0308
G5	307929	310211	2280	7,94	83,90	G5Q_0296
G6	327859	330555	2694	5,79	95,08	G5Q_0307
G7	313712	316234	2520	6,50	90,12	G5Q_0299
G8	310741	312858	2115	6,77	75,81	G5Q_0297
H	301585	304539	2952	7,06	105,05	G5Q_0293

^a^ The *Chlamydia psittaci* Cal10 *pmpG* sequences were used to search for *pmpG* sequences across the collection of isolates currently in the NCBI database. All hits were parsed, and protein sequences were aligned using ClustalW. The alignments were used to manually subdivide the pmpG family into subfamilies.

^b^ The theoretical Mw includes the signal sequence.

### Transcription of *pmp* CDSs

**Fig 2:**
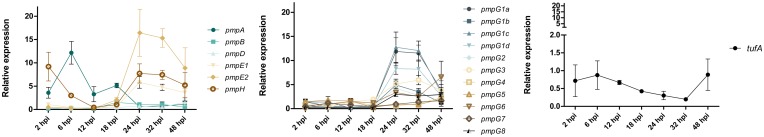
Transcription of the *pmp* genes along development. Transcript levels of *pmpA* through *pmpG8* and *tufA* of *C*. *psittaci* Cal10 were measured at 2, 6, 12, 18, 24, 32 and 48 hpi by real-time RT-qPCR. Two biological and two technical replicated were analyzed for each sample. Error bars are based on the standard error of the mean.

To start unravelling Pmp function, we first determined the timing of Pmp production during chlamydial development. To this end, *pmp* transcript levels were measured under normal culture conditions at early (2 and 6 hours hpi), mid (12 and 18 hpi), and late (24, 32 and 48 hpi) stages of the developmental cycle (Fig 2). Analysis at very late developmental times (e.g., 72 hpi) was not possible because of gradual loss of viability and lysis of the host cells. The gene *tufA* (encoding Elongation Factor Tu, EF-Tu) was included for comparative purposes as a house-keeping gene presumed to be expressed throughout the developmental cycle [31]. Unexpectedly, transcript levels for *tufA* peaked at 6 hpi, then gradually diminished until 32 hpi, and rose again at 48 hpi. Overall, all *pmp* CDSs were transcribed at a detectable level at some stage of the developmental cycle, consistent with observations of *pmp* transcription in other *Chlamydia* species [29–31]. All *pmp* genes, were transcribed at very low levels early in the developmental cycle (2 hpi) except *pmpA* and *pmpH*, that were transcribed at or near peak levels. For most *pmp* genes (*pmpE1*, *pmpE2*, *pmpG1a*, *pmpG1b*, *pmpG1c*, *pmpG1d*, *pmpG2*, *pmpG3*, *pmpG6*, *pmpG8*, and *pmpH*), transcript levels were highest at 24 hpi and remained high at 32 and 48 hpi, except for *pmpG3* and *pmpG6* that peaked at 32 and 48 hpi respectively, and *pmpG8* that remained high from 24 hpi onward. Surprisingly, *pmpH* transcription was highest at 2–6 hpi, minimal at 12–18 hpi and rose again to a high level from 24 hpi onward. Somewhat similarly, *pmpA* transcript levels were highest between 2 and 18 hpi, and rose again from 24 hpi onward although to a lower level. *pmpB* and *pmpD* transcription was relatively low, peaking at 18–32 and 48 hpi, respectively. Transcription of the 2 *pmpG* alleles *pmpG4* and *pmpG5*, peaked at 48 hpi.

### Most *pmp* CDSs of *C*. *psittaci* Cal10 are not co-transcribed

**Fig 3:**
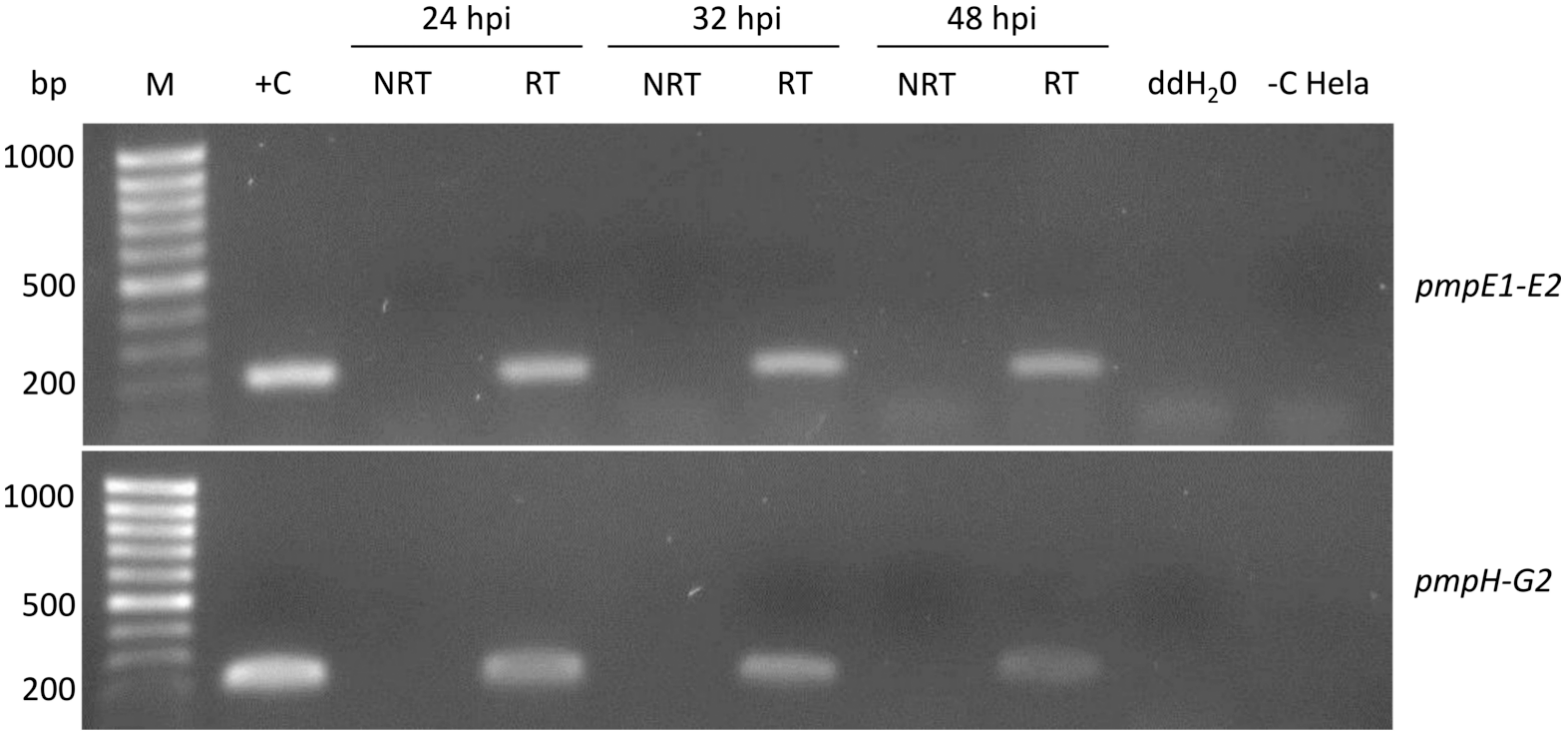
*pmpE1-E2* and *pmpH-G2* are organized in operons. Based on co-linearity, 6 putative operons were identified: *pmpE2-E1*, *pmpG8-G5-G2-H*, *pmpG3-G7*, *pmpG1b-G1d pmpG4-G6-G1c*, and *pmpA-B*. cDNAs generated at 24, 32 and 48 hpi were amplified by RT-PCR using specific primers spanning the intergenic regions (Table 2). Only results for *pmpE1-E2* and *pmpH-G2* are presented as amplicons were not detected for all other intergenic regions. M: MassRuler Low Range DNA ladder (Thermo Scientific); +C: positive control *C*. *psittaci* Cal10 genomic DNA; -C HeLa: negative control cDNA from uninfected HeLa cells; RT/NRT: with or without reverse transcriptase.

The genetic linkage and colinearity of *pmpG4-G6-G1c*, *pmpG1b-G1d*, *pmpG3-G7*, *pmpG8-G5-G2-H*, *pmpE2-E1 and pmpA-B* (Fig 1) suggested that these CDSs might be organized in operons, leading to co-transcription. To partially test this hypothesis, we performed RT-PCR using primers designed to span the relevant intergenic regions (Table 1) on cDNA samples generated during normal cell culture conditions at 24, 32 and 48 hpi. The latter times were selected as the *pmp* genes of *C*. *trachomatis*, *C*. *pneumoniae* and *C*. *abortus* were highly transcribed at mid and late time points [29–31]. Only intergenic regions between *pmpE1-E2* and *pmpH-G2* were amplified (Fig 3), suggesting that *pmpE1-E2* and *pmpG2-H* are arranged in operons, whereas that *pmpG4-G6*-*G1c*, *pmpG1b*-*G1d*, *pmpG3*-*G7*, *pmpG8*-*G5*, and *pmpA-B* are not.

### Protein production profiles differ among *C*. *psittaci* Pmps

**Fig 4:**
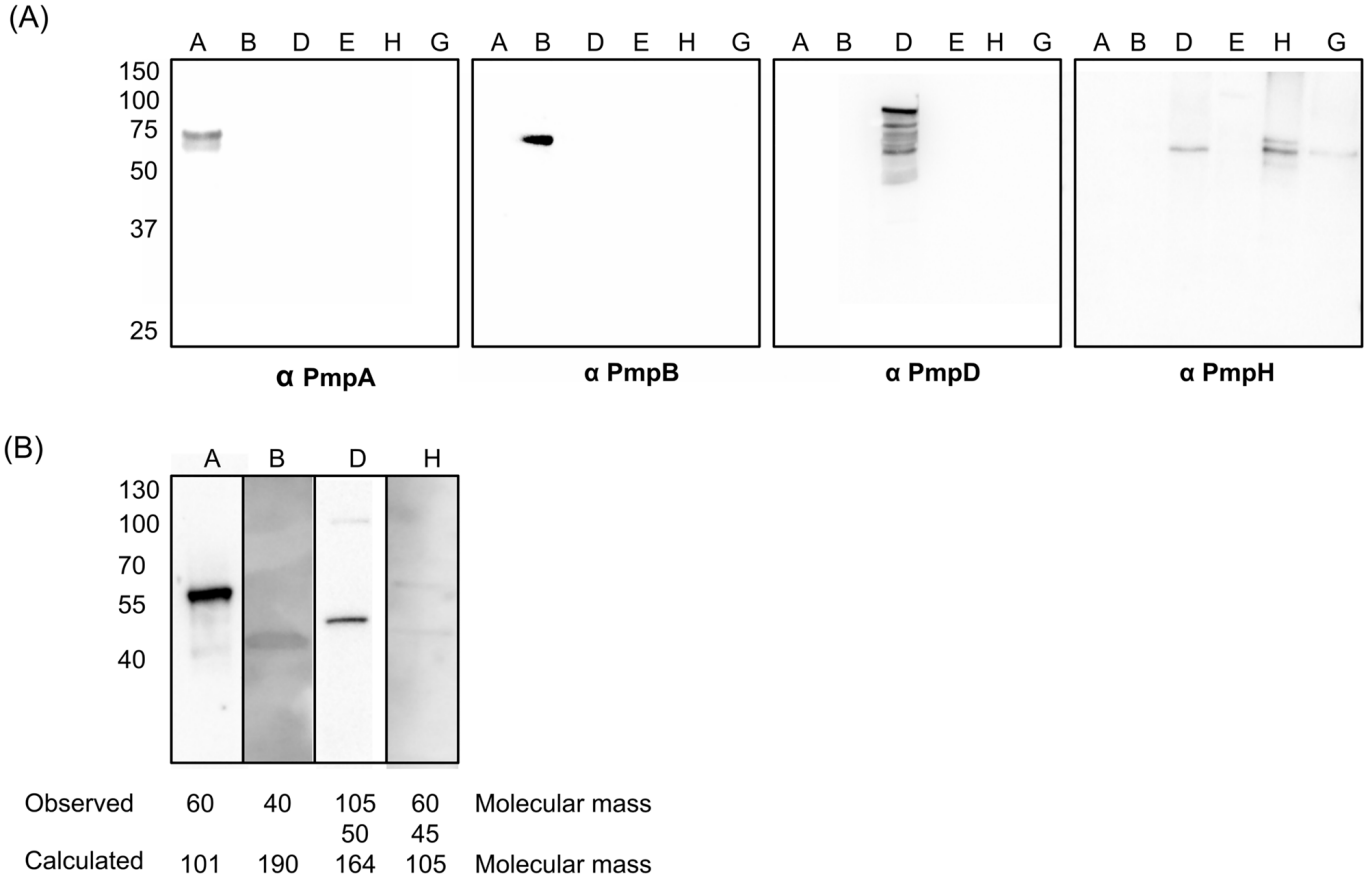
Guinea pig polyclonal antibodies against PmpA, B, D and H are specific for their respective immunizing antigens. (A) The specificity of polyclonal antibodies raised against rPmpA (anti-PmpA), rPmpB (anti-PmpB), rPmpD (anti-PmpD) and rPmpH (anti-PmpH) was verified by immunoblotting using (A) partially purified recombinant PmpA, B, D, E1, G3 and H as well as (B) density gradient purified EBs of *C*. *psittaci* Cal10. The calculated molecular masses of recombinant PmpA, PmpB, PmpD, PmpE1, PmpH and PmpG3 are 92 kDa, 74 kDa, 95 kDa, 74 kDa, 88 kDa and 60 kDa, respectively. The observed molecular masses were 75 kDa, 74 kDa, 95 kDa, 70 kDa, 75 kDa and 60 kDa, respectively. (B) The calculated and observed molecular masses of the protein bands detected in EBs are shown.

RT-qPCR profiles provide an indication of when and how strongly the *pmp* genes are transcribed at the population level. To assess expression at the protein level in individual inclusions, polyclonal, monospecific antibodies against PmpA, PmpB, PmpD, PmpE1, PmpG3, and PmpH were generated in guinea pigs. Analysis of the specificity of the antisera by immunoblot against a panel of rPmps and gradient-purified *C*. *psittaci* EBs indicated that PmpA-, PmpB-, PmpD- and PmpH-specific antibodies reacted with high molecular mass bands (Fig 4A) in the corresponding immunizing antigen lane, while PmpE1- and PmpG3-specific antibodies cross-reacted with multiple bands in multiple lanes (not shown). Therefore, PmpA-, PmpB-, PmpD- and PmpH-antisera were selected for further analysis. The observed molecular masses of rPmpA and rPmpH were smaller than the calculated molecular masses of the corresponding recombinant polypeptides. This may be caused by the instability of the full-length recombinant polypeptides. rPmpH was detected as a triplet of bands of 75, 70 and 60 kDa. However, the 70kDa band was also detected in the rPmpD and rPmpG3 lanes, suggesting that it corresponds to an *Escherichia coli* cross-reactive contaminant. Antibody-reactive bands detected in Western blots against purified EB proteins were also smaller than the calculated molecular masses of each Pmp (Fig 4B). Thus, the Pmps are probably proteolytically processed or degraded, as suggested for *C*. *trachomatis* [35,36] or they are too unstable to survive the EB purification procedure.

**Fig 5:**
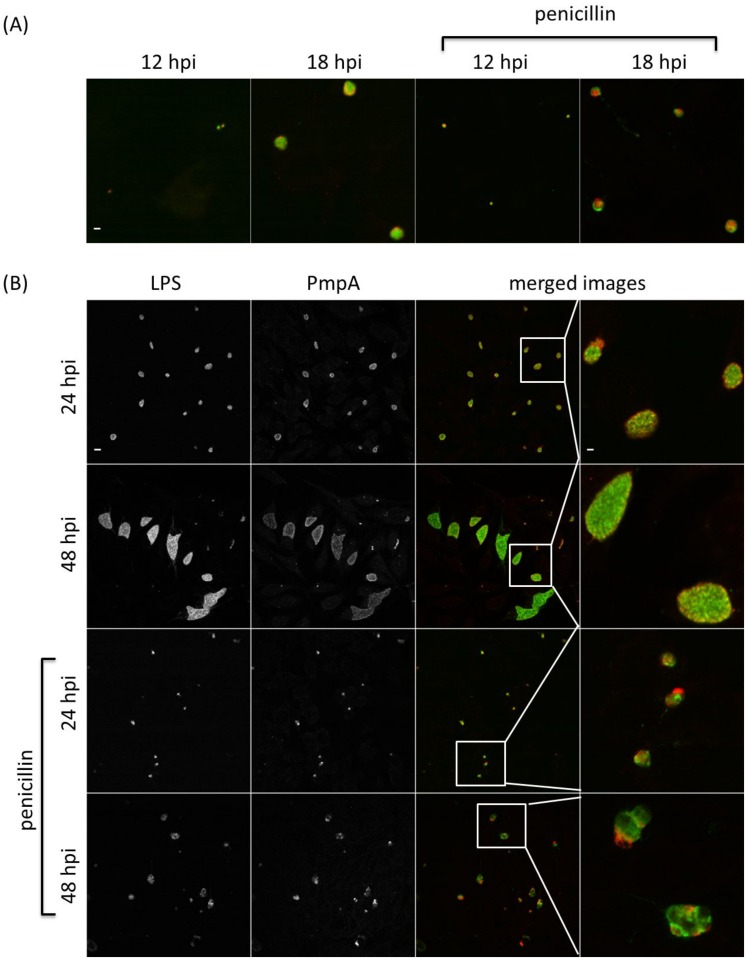
The PmpA production profile along development. *C*. *psittaci*-infected HeLa cells were fixed at 12, 18, 24, 32 and 48 hpi, and double-stained with chlamydial LPS-specific antibody (FITC-conjugated, green) and PmpA-specific antibody (Alexa Fluor 568-conjugated, red). At 12 and 18 hpi (A), only colored merged images are shown under normal and penicillin-induced persistence culture conditions. At 24 and 48 hpi (B), single channel images are shown in black and white (2 left-most columns), while merged images and insets thereof are shown in color (2 right-most columns). Staining patterns at 32 hpi (not shown) were similar to those at 48 hpi. Bar = 2 μm for the 12 and 18 hpi times, and 24 and 48 hpi insets (i.e. top row and right-most column) and 10 μm for all remaining images.

**Fig 6:**
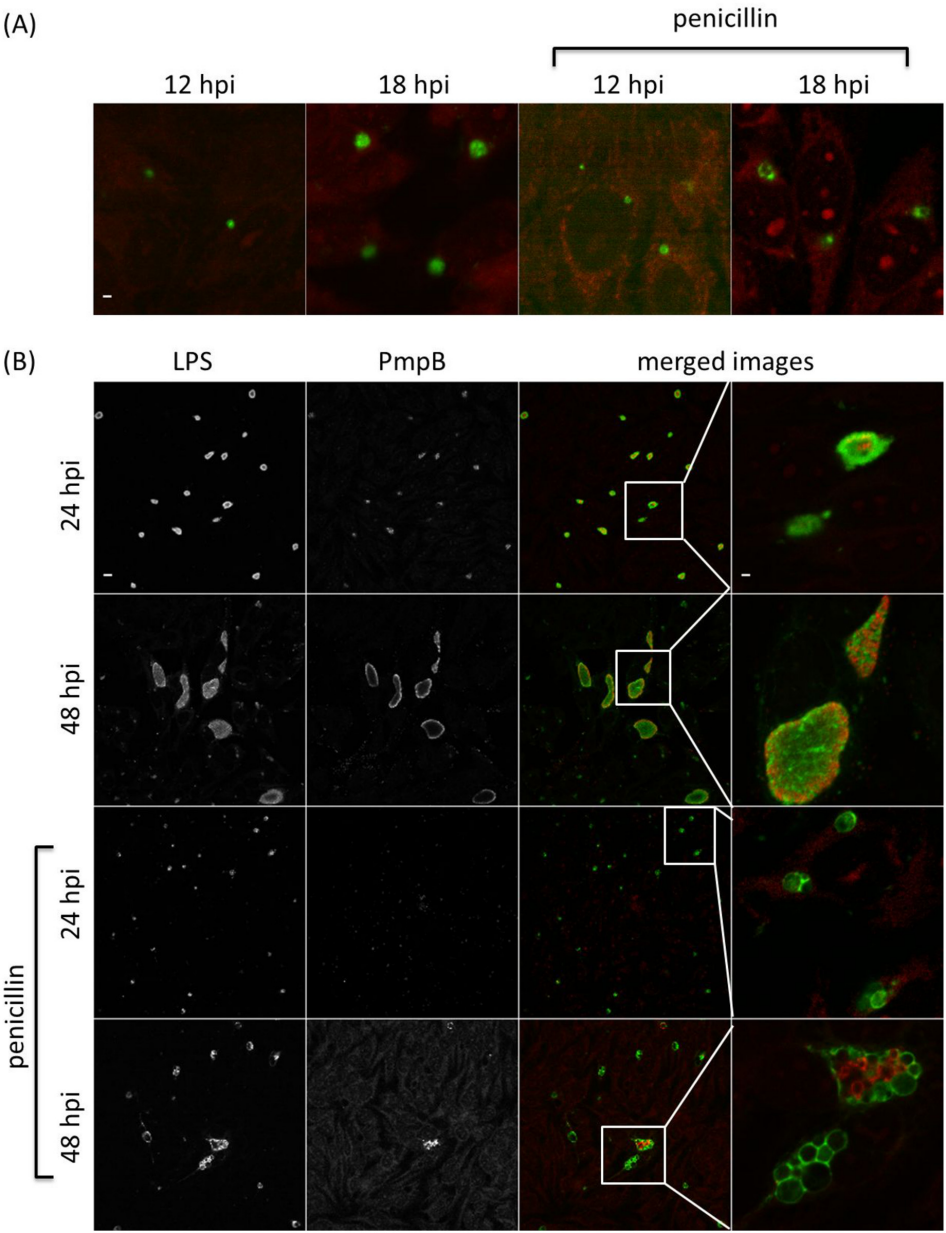
The PmpB production profile along development. *C*. *psittaci*-infected HeLa cells were fixed at 12, 18, 24, 32 and 48 hpi, and double-stained with chlamydial LPS-specific antibody (FITC-conjugated, green) and PmpB-specific antibody (Alexa Fluor 568-conjugated, red). At 12 and 18 hpi (A), only colored merged images are shown under normal and penicillin-induced persistence culture conditions. At 24 and 48 hpi (B), single channel images are shown in black and white (2 left-most columns), while merged images and insets thereof are shown in color (2 right-most columns). Staining patterns at 32 hpi (not shown) were similar to those at 48 hpi. Bar = 2 μm for the 12 and 18 hpi times, and 24 and 48 hpi insets (i.e. top row and right-most column) and 10 μm for all remaining images.

**Fig 7:**
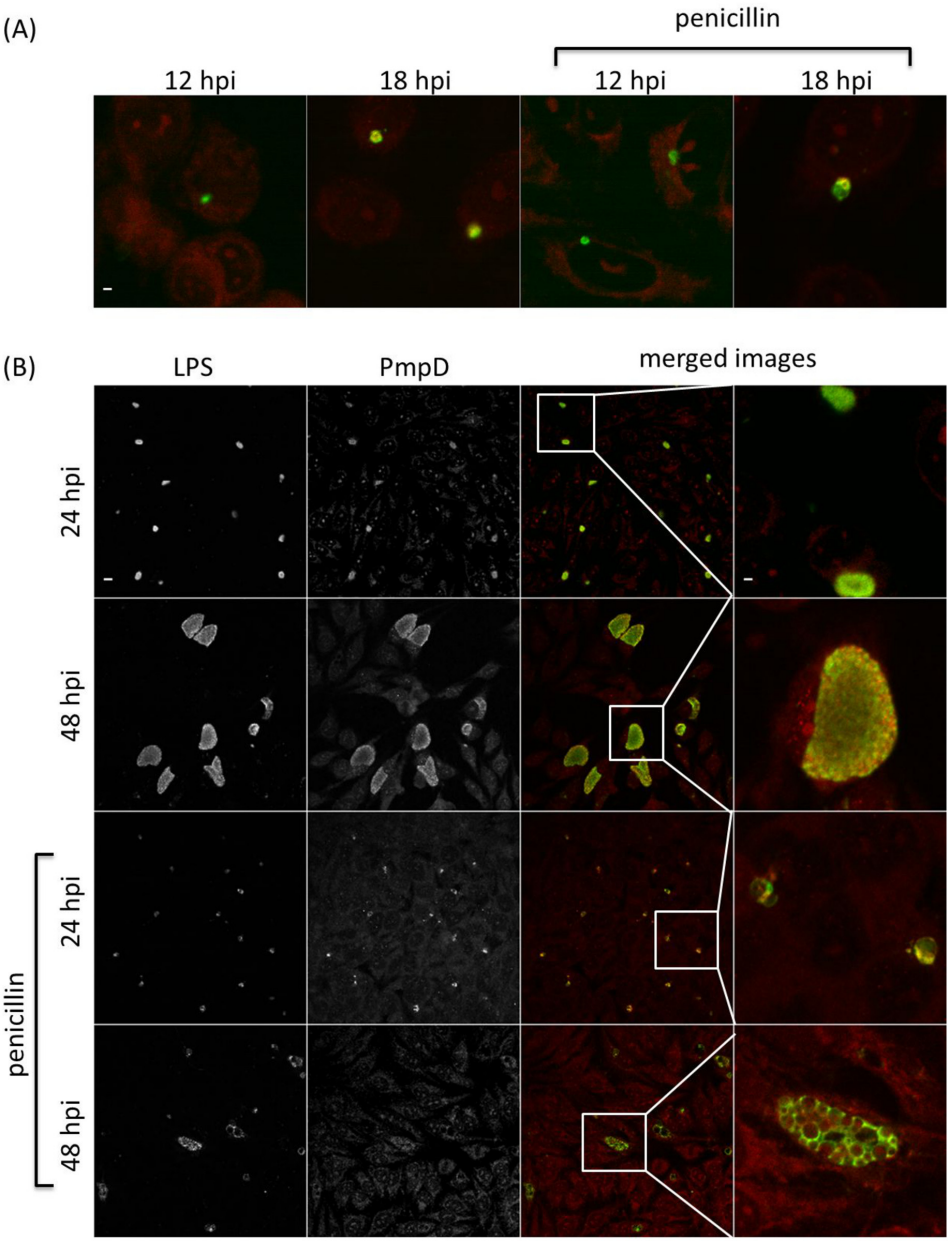
The PmpD production profile along development. *C*. *psittaci*-infected HeLa cells were fixed at 12, 18, 24, 32 and 48 hpi, and double-stained with chlamydial LPS-specific antibody (FITC-conjugated, green) and PmpD-specific antibody (Alexa Fluor 568-conjugated, red). At 12 and 18 hpi (A), only colored merged images are shown under normal and penicillin-induced persistence culture conditions. At 24 and 48 hpi (B), single channel images are shown in black and white (2 left-most columns), while merged images and insets thereof are shown in color (2 right-most columns). Staining patterns at 32 hpi (not shown) were similar to those at 48 hpi. Bar = 2 μm for the 12 and 18 hpi times, and 24 and 48 hpi insets (i.e. top row and right-most column) and 10 μm for all remaining images.

**Fig 8:**
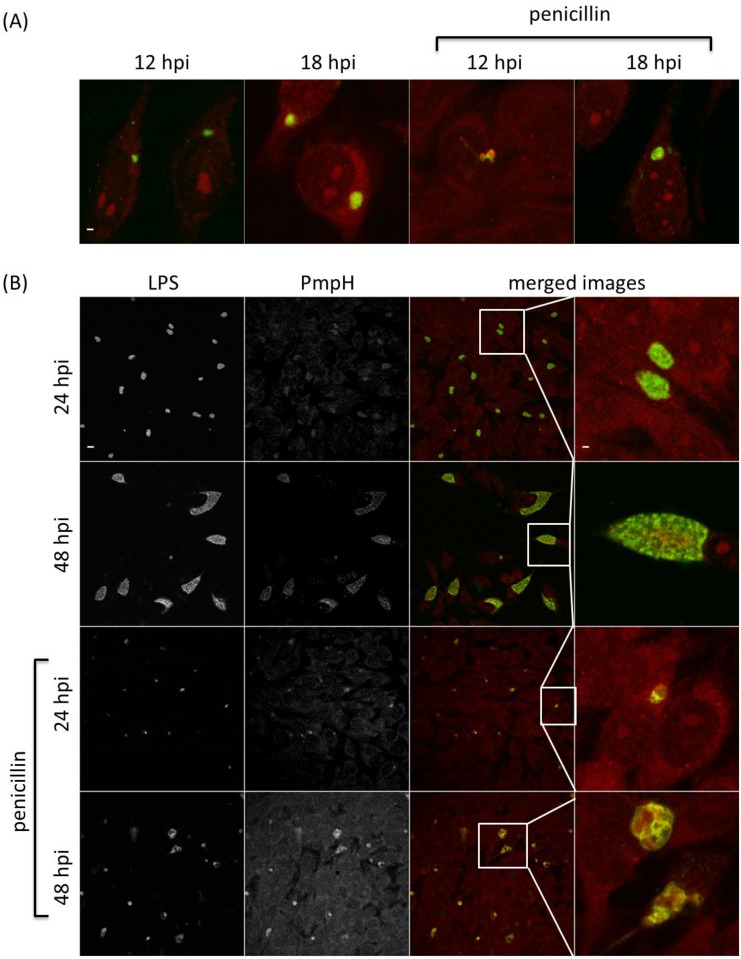
The PmpH production profile along development. *C*. *psittaci*-infected HeLa cells were fixed at 12, 18, 24, 32 and 48 hpi, and double-stained with chlamydial LPS-specific antibody (FITC-conjugated, green) and PmpH-specific antibody (Alexa Fluor 568-conjugated, red). At 12 and 18 hpi (A), only colored merged images are shown under normal and penicillin-induced persistence culture conditions. At 24 and 48 hpi (B), single channel images are shown in black and white (2 left-most columns), while merged images and insets thereof are shown in color (2 right-most columns). Staining patterns at 32 hpi (not shown) were similar to those at 48 hpi. Bar = 2 μm for the 12 and 18 hpi times, and 24 and 48 hpi insets (i.e. top row and right-most column) and 10 μm for all remaining images.

**Fig 9:**
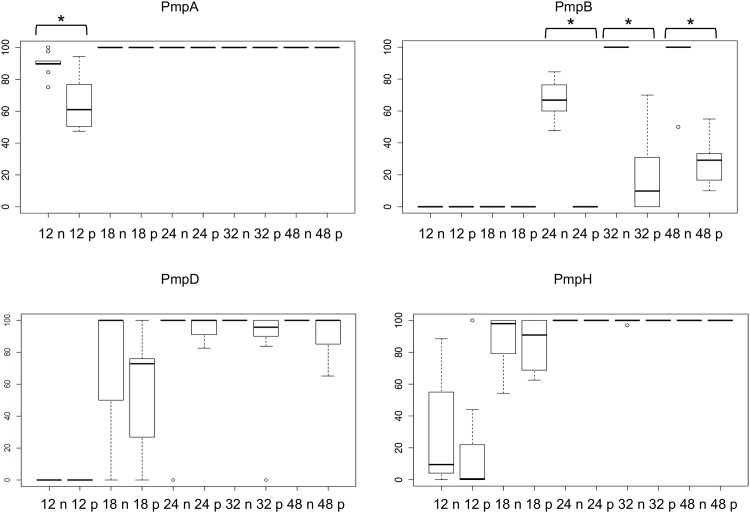
PmpA, B, D and H production differs under normal *C*. *psittaci* and during penicillin-induced stress culture conditions. *C*. *psittaci* infected HeLa cells were fixed at 12, 18, 24, 32 and 48 hpi, and double-stained with a chlamydial LPS-specific antibody and Pmp-specific antibody. For each Pmp subtype, the percentage of positive inclusions was determined by a macro based on co-localization of the two antigens. The results shown here did not take into account smaller inclusions that are formed at 24, 32 and 48 hpi. The data are expressed as box plots: the box represents the 25^th^-75^th^ percentiles, the median is depicted by a bar across the box and the whiskers on each box represent minimum and maximum value. Outliers are depicted by dots. Statistically significant differences (P < 0.05) are indicated with an asterix.

Immunofluorescence (IF) was used to investigate the PmpA, PmpB, PmpD and PmpH protein production profiles in individual inclusions in 10 biological replicates, and to examine potential post-transcriptional and post-translational regulation. Inclusions positive for a specific Pmp subtype were determined under normal *C*. *psittaci* culture conditions and during penicillin-induced stress [31]. The same time points as used for the RT-qPCR were analyzed. At 24 and 48 hpi, both large size and smaller, ovoid and irregularly shaped inclusions were observed (Figs 5–8) during normal culture conditions, possibly the result of the asynchronous start and/or growth of these inclusions. A macro excluding the smaller inclusions was used to quantify the percentage of inclusions producing PmpA, PmpB, PmpD and PmpH, as the large inclusions are representative for the late developmental times (Fig 9).

At 12 hpi, under normal *C*. *psittaci* culture conditions, PmpA was highly produced in all 10 biological replicates, with a median of 90% PmpA positive inclusions (Fig 9), consistent with transcription results (Fig 2). At 12 hpi, PmpH was not equally produced in all replicates, such that in 3 replicates, PmpH was detected in 88% of the inclusions, while in 7 replicates, it was only detected with a median of 4% of the inclusions (Fig 9). Thus, PmpH production displayed substantial variation between cultures inoculated with the same seed. Neither PmpB- nor PmpD-positive inclusions were detected at 12 hpi and PmpB-positive inclusions were also not detected at 18 hpi (Figs 6, 7 and 9). At 24, 32 and 48 hpi, PmpA and PmpH were highly expressed in all replicates with a median percentage of positive inclusions of 100% (Figs 5, 8 and 9). PmpB was first detected at 24 hpi in a median of 67% of the inclusions and was highly expressed in all inclusions at 32 and 48 hpi (Figs 6 and 9). Noticeably, the expression of PmpD varied significantly between biological replicates at 18 and 24 hpi, as PmpD was not detectably produced in 1 replicate at both times and between 88% and 99% in the other 9 replicates (Fig 9).

### Penicillin-induced stress alters protein production profiles of specific *C*. *psittaci* Pmps

Because of their obligate intracellular life style, the pathogenesis of chlamydiae is intimately linked to their capacity to grow inside cells and on their specific physiologic properties in response to threats such as innate host defenses or nutrient deprivation. To start evaluating potential differential production of Pmp subtypes under different growth conditions, we used penicillin-induced stress as a previously well-defined modulator of *pmp* gene expression in *C*. *trachomatis* [31]. Under penicillin-induced stress, PmpA was the only detectable Pmp at the early developmental time of 12 hpi (Figs 5–9). PmpA production was observed in all 10 biological replicates, yielding a median of 61% PmpA positive inclusions, which is significantly lower than the 90% observed under normal conditions (Fig 9). At the same developmental time, PmpH was unevenly expressed with 100% of the inclusions staining positive for PmpH in 1 biological replicate but only 0.3% in the other nine, yielding a median of 0.5% PmpH-positive inclusions (Fig 9). PmpB and PmpD, which were not expressed at 12–18 hpi, and 12 hpi respectively in unstressed cultures, were also not detected at these times during penicillin-induced stress (Figs 6, 7 and 9).

At 18, 24, 32 and 48 hpi, PmpA, PmpD and PmpH were expressed in all biological replicates experiencing penicillin-induced stress at levels at or near those of unstressed cultures (Fig 9). The median percentage of Pmp positive inclusions was 100% for PmpA at all time points, and 91, 100, 100 and 100% at 18, 24, 32 and 48 hpi respectively for PmpH (Fig 9). Relatively fewer PmpD-positive inclusions (59%) were observed at 18 hpi, i.e. significantly less than for PmpA and PmpH (P < 0.05) while 93, 84 and 88% of the inclusions were PmpD-positive at 24, 32 and 48 hpi, respectively, under penicillin-induced stress (Fig 9). Interestingly, at 18 and 32 hpi, the level of PmpD expression also varied among the biological replicates experiencing penicillin-induced stress, with no expression in 1 replicate, and 65 to 88% respectively in the remaining nine (Fig 9). PmpB expression was significantly down-regulated at all developmental times during penicillin-induced stress (Fig 9). It is noteworthy that the relative number of inclusions producing PmpB, PmpD or PmpH rose significantly (P < 0.05) when the smaller inclusions were omitted from the analyses. However, this was not the case for PmpA, highlighting the very high level of expression of PmpA at early stages of the *C*. *psittaci* developmental cycle.

### PmpA, PmpD and PmpH target different subcellular sites

**Fig 10:**
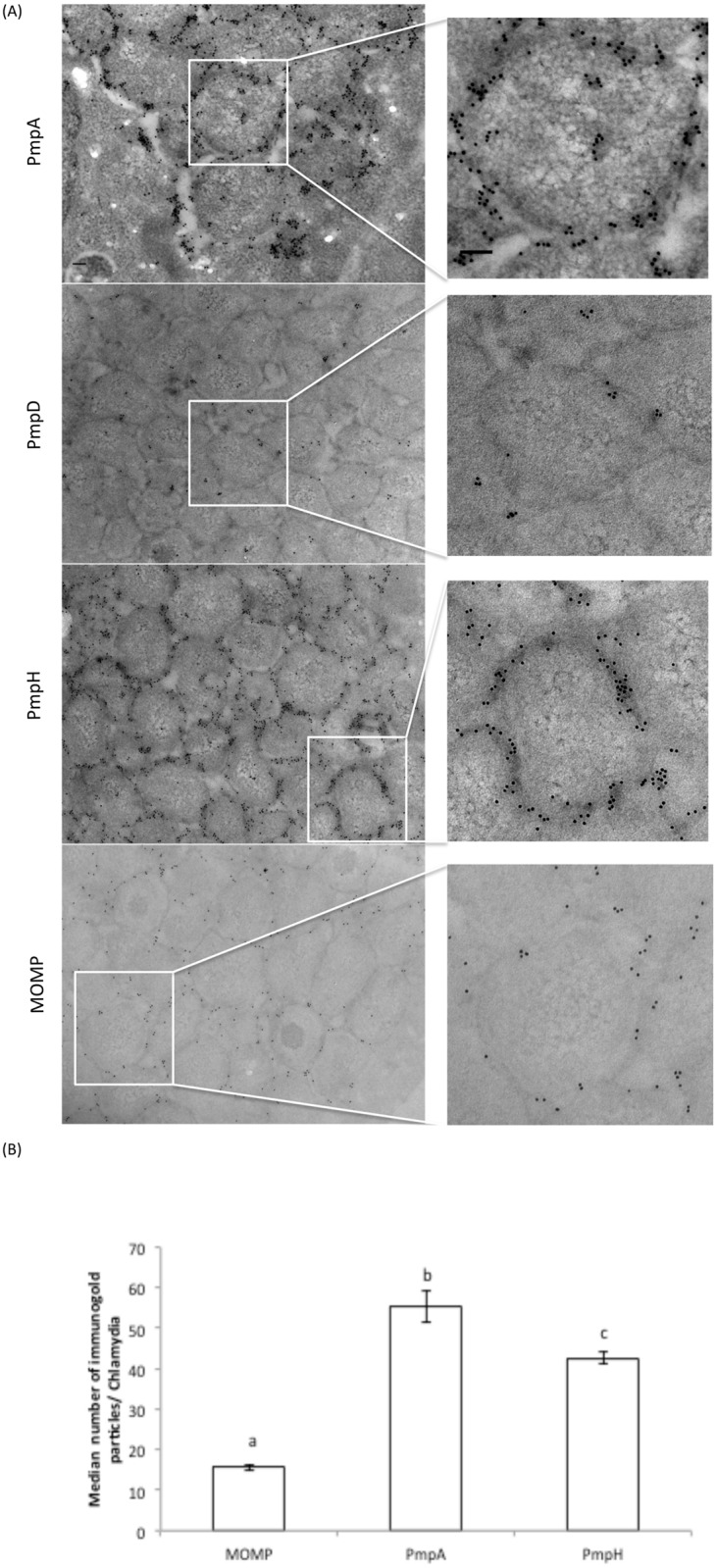
PmpA and PmpH stain more heavily in the chlamydial envelope than MOMP. (A) *C*. *psittaci* infected HeLa cells were fixed at 24 hpi and stained with primary Pmp-specific antibody and secondary gold conjugated goat anti-guinea pig antibody. Subcellular localization did not change at 48 hpi (not shown). Bars = 0.1 μm. (B) *C*. *psittaci* infected HeLa cells were fixed 24 hpi, labeled with MOMP-, PmpA- and PmpH- specific antibodies and secondary gold conjugated goat anti-rabbit or gold conjugated goat anti-guinea pig antibody and the number of immunogold particles was counted on 100 chlamydiae. Error bars are based on standard error of the mean. Different letters indicate statistically significant differences (P < 0.05).

Immuno-electron microscopy (IEM) was used to assess the subcellular localization of Pmp proteins at late developmental times. Probably due to compromised antigen recognition or accessibility during dehydration and fixing for IEM, PmpB staining was poor preventing further analysis of this Pmp subtype by this method. Consistent with RT-qPCR and IF results, IEM revealed strong PmpA-specific signal mostly localized at the chlamydial cell envelope (Fig 10A). PmpH IEM staining was similar to that of PmpA in localization and relative abundance. PmpD-specific labeling was also observed at the chlamydial cell envelope, but less abundant than PmpA and H (Fig 10A).

**Fig 11:**
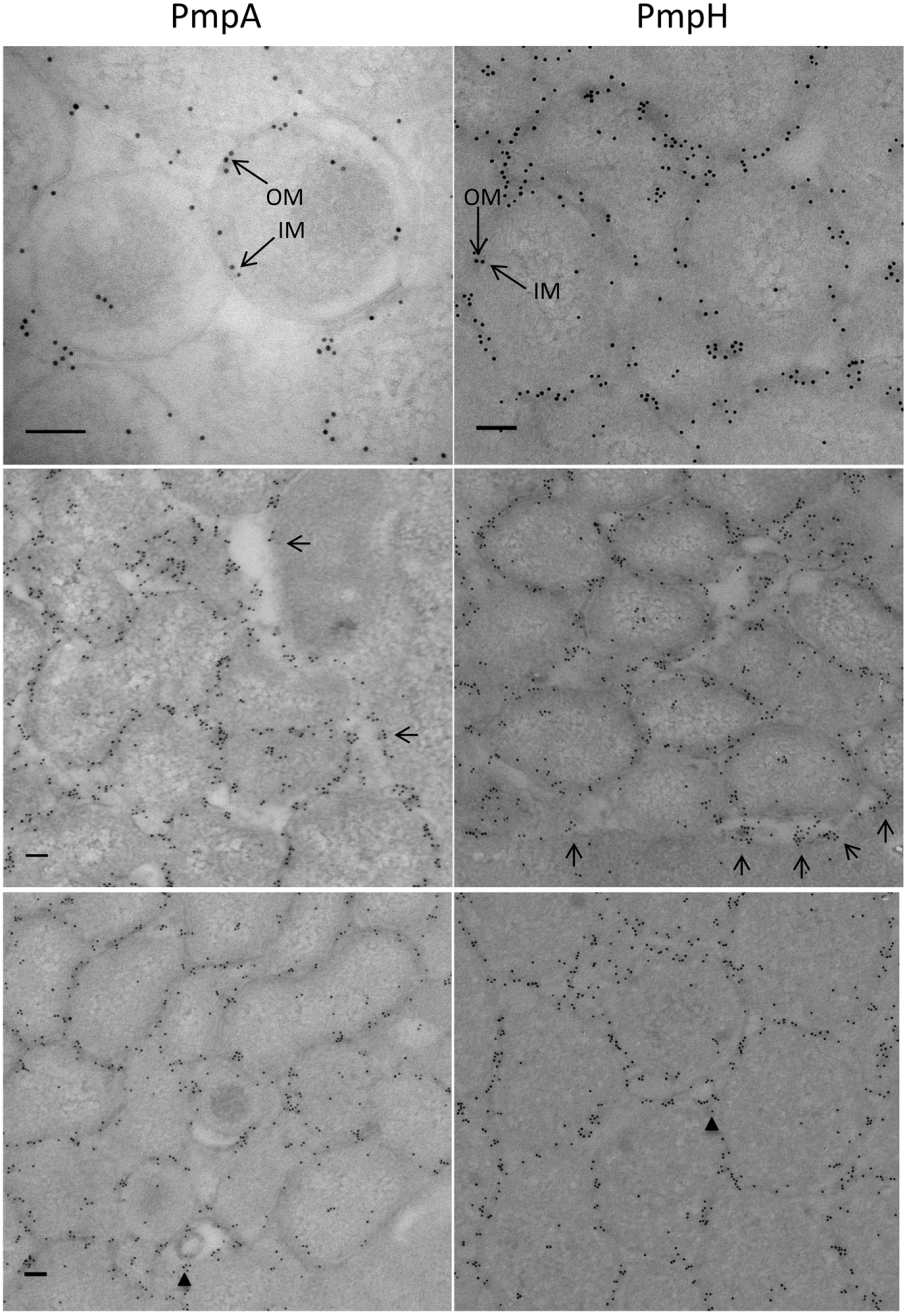
PmpA and PmpH localize to the chlamydial inner and outer membranes, to the inclusion membrane and to small putative outer membrane vesicles. *C*. *psittaci* infected HeLa cells were fixed at late times (24 and 48 hpi) and stained with primary PmpA- or PmpH-specific antibody and secondary gold conjugated goat anti-guinea pig antibody. Localization of PmpA and PmpH in the chlamydial inner (IM) and outer (OM) membranes (top row, long arrows), in the inclusion membrane (middle row, short arrows) and in putative OMVs (bottom row, arrowheads) is shown as indicated. Bars = 0.1 μm.

To evaluate relative antigenicity and immuno-accessibility, we compared IEM staining of MOMP, the most abundant chlamydial protein and a strong antigen during infection in other *Chlamydia* spp. [46,47], with that of *C*. *psittaci* PmpA and PmpH at 24 hpi. Surprisingly, PmpA and PmpH were significantly more labeled at the cell envelope than MOMP (Fig 10A and 10B). PmpA was also significantly more labeled than PmpH. Detailed examination of higher magnification images revealed both inner and outer membrane labeling of all analyzed Pmps and inclusion membrane labeling with PmpA-, and PmpH-specific antibodies (Fig 11). PmpA-, and PmpH-specific staining of small vesicles, possibly corresponding to outer membrane vesicles (OMVs), was also observed at 48 hpi (Fig 11).

## Discussion

An ultimate assessment of the involvement of the Pmp family in *C*. *psittaci* virulence and their potential use as vaccine candidates requires the fundamental characterization of the expression and topology properties of these proteins. We used *C*. *psittaci* Cal10, a previously well-characterized, prototypic strain [48–50], with a complete genome sequence [51]. The number of *pmp* CDSs identified in the *C*. *psittaci* Cal10 genome differs from the 21 *pmp* CDSs that were identified by Voigt *et al*. [52] in the *C*. *psittaci* 6BC genome. The number of *pmp* CDSs is not only different across *Chlamydia* species but also within a *Chlamydia* species (not shown). Consistent with observations of *pmp* gene expression in other *Chlamydia* species [29–31], transcripts were detectable for all 17 *C*. *psittaci pmp* CDSs, although the level of transcription varied over a wide range among *pmps* and with developmental time for each *pmp*. Transcript levels were consistently high for *pmpA*, the co-transcribed *pmpE1* and *pmpE2*, *pmpH* and several *pmpG* alleles (e.g., G1a, G1c), and consistently low for *pmpB*, *pmpD* and other *pmpG* alleles (e.g., *pmpG4*, *pmpG5*). Moreover, while nearly all *pmp* transcript levels were high at late developmental times (24–48 hpi; typically with a dip at 48 hpi), *pmpA* transcript levels were high at early-to-mid developmental times (2–18 hpi), similar to the *pmpA* ortholog of *C*. *trachomatis*, but opposite to the late expressed *pmpA* ortholog of *C*. *abortus* [30,31]. Transcript levels for *pmpH* stood out in that they were high at early (2–6 hpi), low at mid (12–18 hpi) and high again at late developmental times (24–32 hpi). This feature appears to be unique for *pmpH* of *C*. *psittaci* as in all other species, *pmpH* is characteristically transcribed late [30,31] and suggests PmpH plays an important role at both ends of the *C*. *psittaci* developmental cycle. It also indicates that *pmpH* transcript is relatively unstable during mid-cycle development. These observations and the additional observation that *pmpH* is co-transcribed with *pmpG2* suggest that the regulation of *pmpH* expression may follow a complex, multi-level mechanism, and may betray a key role of PmpH in overall Pmp function in *C*. *psittaci*.

Late transcription of most *pmp* genes is in general agreement with the results of *pmp* transcription analyses for *C*. *abortus* and *C*. *trachomatis* where all *pmp* genes, with the exception of *pmp5E* for *C*. *abortus* and *pmpA* and *I* for *C*. *trachomatis*, were differentially highly expressed during late development [30,31]. It is also consistent with transcription of the *pmp* gene family in *C*. *pneumoniae*, where transcripts could be detected for all known *pmp* genes at 72 hpi [29]. Globally, transcription analyses of *pmp* gene families across several *Chlamydia* species suggests an important role for these proteins either at late stages of development or, upon storage of late-expressed Pmps in EBs during the early steps of infection. Conversely, differences at the species level (e.g. *pmpA* and *pmpH* of *C*. *psittaci* Cal10 versus orthologs in *C*. *trachomatis* and *C*. *abortus*) may reveal functional differences that are intrinsic to the properties of each species.

Transcription of the expended 11-member *pmpG* family of *C*. *psittaci* revealed an intriguing feature: while all *pmpG* alleles appeared to be transcribed to some level at late developmental times, the level of peak transcription varied almost linearly from the high of *pmpG1a/c/d*, and in decreasing order *pmpG3/6/1b*, *pmpG2/8* to the relatively low levels of *pmpG4/5/7*. Low levels transcripts were similar or slightly higher than those for the reference gene *tufA*, a gene required for exponential growth [31] suggesting that the relatively low levels of *pmp* transcripts detected by RT-qPCR represent actual functional transcript levels, i.e. not experimental background. This is further supported by evidence of Pmp protein production for some of the more weakly transcribed *pmp* genes (e.g., *pmpD*; see below). Interestingly, the *pmpG1c* and *pmpG1d* alleles were independently transcribed at high levels. These alleles putatively encode truncated Pmp polypeptides devoid of the autotransporter domain suggesting that they would not be secreted to the chlamydial surface if expressed. These observations highlight the limitations of transcriptional analysis for functional studies of mixed genomic populations [53,54], particularly as it concerns the *pmp* gene family. To address this issue, we investigated *pmp* expression at the protein level, using immunofluorescence (IF) and immuno-electron microscopy (IEM) to determine Pmp production respectively at the inclusion and at the chlamydial cell levels, in multiple biological replicates. Anti-PmpA, anti-PmpB, anti-PmpD and anti–PmpH pAbs reacted specifically with the corresponding recombinant protein by immunoblot analyses. Immunoblot analyses against gradient-purified *C*. *psittaci* EBs demonstrated that anti-PmpH sera reacted specifically with high molecular mass bands, however, those bands were faint. Grimwood *et al*. [29] found that PmpH is susceptible to proteolysis. Therefore, we suggest that PmpH might be degraded after EB purification.

The early high-level *pmpA* transcription was matched by high-level production of PmpA during all stages of development in all biological replicates, indicating the relative stability of the *pmpA* mRNA and PmpA protein and suggesting a relative absence of *pmpA* variation at the genomic level in the study population (the *C*. *psittaci* culture). PmpB, for which the encoding gene was relatively poorly transcribed, was detectably produced late in the majority of inclusions in all replicates. Production of PmpD was also detected at developmental times generally corresponding to maximum transcription but was undetectable in one of ten biological replicates at 18 and 24 hpi. This may be loosely comparable to the on-off phase-like variation described for *pmpD* of *C*. *trachomatis* [36]. PmpH, whose transcript exhibited discontinuous high-low-high levels during development, was strongly produced late in all replicates, but only in some of the replicates at mid developmental cycle, possibly owing to different translation start times for this protein in different cultures.

Multiple types of stress have been shown to affect chlamydial growth and morphology and to down- or up-regulate various chlamydial genes important in virulence [55–57]. We used penicillin-induced stress to comparatively investigate Pmp protein production in *C*. *psittaci* inclusions. Similar to its ortholog in *C*. *trachomatis* [31], production of PmpA was not significantly affected by penicillin-induced stress. PmpB production was also similarly down regulated in *C*. *psittaci* and *C*. *trachomatis*. PmpD and PmpH productions however, whose orthologs are down-regulated in *C*. *trachomatis* [31], were unaffected by penicillin in *C*. *psittaci*. This may again highlight a yet-to-be-determined species-specific function of PmpD and PmpH in *C*. *psittaci*.

IEM was used to assess the subcellular localization of the Pmps. PmpA, D and H localized to the *C*. *psittaci* cell envelope (both outer and inner membrane labeling). This is consistent with the observed surface localization of all nine Pmps of *C*. *trachomatis* [27,35,36], Pmp6, 8, 10, 11, 18 and 21 of *C*. *pneumoniae* (orthologs of PmpGs [Pmp6, 8, 10 and 11], PmpE and PmpD of *C*. *trachomatis*, respectively) [21,22] and the 90kDa Pmp (ortholog of PmpG of *C*. *trachomatis*) of *C*. *abortus* [20]. Remarkably, we observed more abundant antibody labeling of PmpA and PmpH at the chlamydial cell envelope than that of MOMP, the major surface-accessible protein in all *Chlamydia* species. Although this observation may owe to differential properties of the antibodies or post-embedding immunolabeling techniques used in our analysis, it may also relate to the unusual antigenicity and immuno-accessibility of these 2 proteins in *C*. *psittaci*. Similar abundant Pmp antibody labeling at the cell envelope has not been observed for *C*. *trachomatis* and *C*. *pneumoniae*, which are phylogenetically relatively distant from *C*. *psittaci* [58]. However, the 90kDa Pmp of *C*. *abortus*, a close phylogenetic relative of *C*. *psittaci*, was also abundantly immunolabeled at the chlamydial surface [20]. We also detected PmpA-, and H-specific immunogold-labeling of small vesicles within the inclusion and of the inclusion membrane itself, a first among all *Chlamydia* species. We speculate that the observed vesicles may have pinched-off from the outer membrane of reticulate bodies and fused to the inclusion membrane. Similarly, Vanrompay *et al*. [59] observed the small vesicles within the inclusions of four different *C*. *psittaci* strains and suggested that the fusion of these vesicles with the inclusion membrane could account for its labeling by the polyclonal antibodies used in their study. Similar results were also reported by Taraska *et al*. [60], for *C*. *trachomatis* and *C*. *psittaci*. Comparable shedding of vesicular material and the contiguous localization of these vesicles with the vacuolar membrane has also been observed for intracellular protozoan parasites [61,62].

Comparative genomics reveal that the *pmp* gene family is highly polymorphic with a differentially high concentration of single nucleotide polymorphisms (SNPs) at *pmp* genomic loci [63,64]. Moreover analysis of any *Chlamydia* genome sequence at the sub-consensus sequence level reveals that the genomic population is most likely composed of many variants, including point and indel mutants and recombinants, with an overrepresentation at specific genomic sites, including *pmp* loci [53,54]. Transcriptional analysis averages levels of expression for any gene and is based on an annotated genomic sequence that itself underrepresents genetic diversity, it will therefore provide an overview that cannot account for diversity at the inclusion or single bacterium levels in the population. Similar to Opa- and Pil-associated diversity in *Neisseria gonnorhoeae* [65,66], we speculate that Pmp-associated phenotypic diversity may be generated by frequent SNPs and indels at *pmp* loci and possibly by reversible recombination of *pmp* gene fragments from transcriptionally silent CDSs into expressed *pmp* genes. These proposed underlying mechanisms are consistent with the observed *pmp* variable expression at different stages of *C*. *psittaci* development and in different biological replicates observed in this study. They may also be phenotypically comparable with observations of phase variation-like phenotypes in *C*. *trachomatis* [36] and *C*. *abortus*-infected cultured cells [32].

A key function of chlamydial Pmps has been described by Mölleken *et al*. [25] and Becker and Hegemann [26] who demonstrated that Pmp6, Pmp20 and Pmp21 of *C*. *pneumoniae* and all Pmps of *C*. *trachomatis* may function as adhesins during infection. Although our studies of *C*. *psittaci* Pmp proteins did not include studies of their role in adherence, structural similarities suggest that a similar role is likely for *C*. *psittaci* Pmps. However many questions remain unanswered. In particular, the intrinsic redundancy within each chlamydial Pmp family, the high rate of sequence polymorphism at *pmp* genomic loci, the observed phase-like variation at various rates for different Pmp subtypes in different species, and the highly variable regulation of pmp gene expression within a species and between species, are not explained to date. Our study aimed to lay the foundation for future functional analyses of the Pmps of *C*. *psittaci*. Although many similarities exist, clear differences with other species have emerged in cultured cells that may relate to differential pathogenic properties of the different species in their respective hosts. PmpA and PmpH of *C*. *psittaci*, by virtue of their unique expression properties emerge as important players in pathogenesis and their apparent immunoaccessibility/antigenicity suggest their potential in vaccine design. These observations thus provide a strong platform for future studies of the Pmp family of *C*. *psittaci*.

## Supporting Information

S1 Table Primers to check the RNase-free DNase I treatment. (DOCX)

S2 Table Primers used for RT-qPCR analysis. (DOCX)
