# Supplementary material for: Analysis of Polymorphic Membrane Protein Expression in Cultured Cells Identifies PmpA and PmpH of Chlamydia psittaci as Candidate Factors in Pathogenesis and Immunity to Infection
Source: PLoS One. 2016 Sep 15;11(9):e0162392. doi: 10.1371/journal.pone.0162392 (PMC5025070; doi:10.1371/journal.pone.0162392)
Supplement: S1 Table — (DOCX) [file pone.0162392.s001.docx]

**S1 Table. Primers to check the RNase-free DNase I treatment**

| **Gene** | **Primer** | **Primer sequence (5’ to 3’)** | **Amplicon size (bp)** |
| --- | --- | --- | --- |
| 16S rRNA | 16S-QPCR-F | TGTACAAGGCCCGGGAACGTA | 156 |
|  | 16S-QPCR-R | GGCCAGTACAGAAGGTAGCA |  |

|  |
| --- |
