# Supplementary material for: Analysis of Polymorphic Membrane Protein Expression in Cultured Cells Identifies PmpA and PmpH of Chlamydia psittaci as Candidate Factors in Pathogenesis and Immunity to Infection
Source: PLoS One. 2016 Sep 15;11(9):e0162392. doi: 10.1371/journal.pone.0162392 (PMC5025070; doi:10.1371/journal.pone.0162392)
Supplement: S2 Table — (DOCX) [file pone.0162392.s002.docx]

**S2 Table. Primers used for RT-qPCR analysis**

| **Gene** | **Primer** | **Primer sequence(5'-3')** | **Amplicon size (bp)** | **Tm (°C)** | **GC (%)** | **primer concentration (nM)** | **Efficiency (%)** |
| --- | --- | --- | --- | --- | --- | --- | --- |
| *pmpA* | pmpA-1 | GTCGCCAGAGAAGGTGTTCC | 112 | 62,2 | 60,0 | 200 | 85,6 |
|  | pmpA-2 | GGGACAAGAAGCACTCAACCT |  | 60,7 | 52,4 | 200 |  |
| *pmpB* | pmpB-1 | TGCTGCAGCGTTAAGAGTGA | 116 | 56,9 | 50,0 | 200 | 66,9 |
|  | pmpB-2 | CCCTAGGCGGTAGCATTACA |  | 56,4 | 55,0 | 200 |  |
| *pmpD* | pmpD-1 | CCAAGACCCTTTGGATTCACC | 115 | 62,9 | 52,4 | 200 | 95,4 |
|  | pmpD-2 | GGGTATCTTTGCTGGGTCGT |  | 61,3 | 55,0 | 200 |  |
| *pmpE1* | pmpE1-1 | CGTGGTAGTATCGATGGTGGAA | 101 | 56,2 | 50,0 | 200 | 88,1 |
|  | pmpE1-2 | GCAGCTGCAACTCGAACAG |  | 57,0 | 57,9 | 200 |  |
| *pmpE2* | pmpE2-1 | GGGTTGAGTGGAGGGCATT | 112 | 57,6 | 57,9 | 200 | 77,2 |
|  | pmpE2-2 | CATGGAGACGCACCCAAGT |  | 57,7 | 57,9 | 200 |  |
| *pmpH* | pmpH-1 | GCAGCTGGATTATCGCCTGT | 105 | 62,6 | 55,0 | 200 | 86,6 |
|  | pmpH-2 | CGCTAACCTCAATGTCCTTGGT |  | 62,6 | 50,0 | 200 |  |
| *pmpG1a* | pmpG1a-1 | CCTCCAAATCTGAAGGGACA | 119 | 54,3 | 50,0 | 200 | 69,9 |
|  | pmpG1a-2 | GGCAAGGTTACCAGCAGTATCA |  | 57,0 | 50,0 | 200 |  |
| *pmpG1b* | pmpG1b-1 | GATAGCGTGGCTGTTCGAGT | 178 | 60,4 | 55,0 | 200 | 66,8 |
|  | pmpG1b-2 | ATCCCATACTCTCCGCATCA |  | 60,5 | 50,0 | 200 |  |
| *pmpG1c* | pmpG1c-1 | AGAAGGTCCCTCTCCGTCAG | 174 | 60,8 | 60,0 | 200 | 63,3 |
|  | pmpG1c-2 | GGTAGAAGCGATCCCGATGT |  | 61,4 | 55,0 | 200 |  |
| *pmpG1d* | pmpG1d-1 | TGACTGTTGCAGCACTGTCTCT | 128 | 58,7 | 50,0 | 200 | 73,6 |
|  | pmpG1d-2 | CCCTACAGGCGGAGCTATTT |  | 56,7 | 55,0 | 200 |  |
| *pmpG2* | pmpG2-1 | CACCACTAATACGGCGGAAA | 108 | 55,0 | 50,0 | 200 | 81,2 |
|  | pmpG2-2 | GCTGTGATGGCTTTGGCTAC |  | 56,7 | 55,0 | 200 |  |
| *pmpG3* | pmpG3-1 | CGTAAGGATAGGCGCTTTGG | 156 | 61,9 | 55,0 | 200 | 68,2 |
|  | pmpG3-2 | GACATTCGCGGACCTGTAGT |  | 60,1 | 55,0 | 200 |  |
| *pmpG4* | pmpG4-1 | GAGGGTTGCACGTCGATTAG | 171 | 60,7 | 55,0 | 200 | 68,7 |
|  | pmpG4-2 | ATCCATACCGAGGGCTTCA |  | 60,4 | 52,6 | 200 |  |
| *pmpG5* | pmpG5-1 | GCGCCTATATGGCTGATGAA | 177 | 61,1 | 50,0 | 200 | 61,2 |
|  | pmpG5-2 | TGATCAAGATTCCCGTCCTG |  | 61,0 | 50,0 | 200 |  |
| *pmpG6* | pmpG6-1 | TAGAACCCGCATAGACGTTTCC | 131 | 62,5 | 50,0 | 100 | 63,3 |
|  | pmpG6-2 | GGGATATGTGTTAGGAGCCACA |  | 61,1 | 50,0 | 100 |  |
| *pmpG7* | pmpG7-1 | CCCAGAATCCTTCAGAACACAG | 194 | 61,0 | 50,0 | 200 | 66,5 |
|  | pmpG7-2 | GGTGACTACTCTTGGCACGAAA |  | 61,6 | 50,0 | 200 |  |
| *pmpG8* | pmpG8-1 | CTGGAGAGTCCTCCCAGGTT | 111 | 60,6 | 60,0 | 200 | 73,3 |
|  | pmpG8-2 | TATCCGCTACAGGGCAAGTC |  | 60,2 | 55,0 | 200 |  |
| *tufA* | ef-tu-1 | ACATAGCTTGCATCGCCTTC | 125 | 60,4 | 50,0 | 200 | 92,2 |
|  | ef-tu-2 | GATGCCGAGCTTGTAGACTTG |  | 60,0 | 52,4 | 200 |  |
| *16S rRNA* | 16SrRNA-1 | TGTACAAGGCCCGGGAACGTA | 156 | 59,9 | 57,1 | 200 | 95,4 |
|  | 16SrRNA-2 | GGCCAGTACAGAAGGTAGCA |  | 58,0 | 55,0 | 200 |  |
| *tyrS* | tyrS-1 | TGGGACAGGCTTATGGTTTG | 169 | 60,9 | 50,0 | 200 | 96,6 |
|  | tyrS-2 | CGTGCGACTTTAGGCACTTC |  | 61,0 | 55,0 | 200 |  |
| *gidA* | gidA-1 | GATCTCCGGGTTGTTCTTCA | 100 | 60,1 | 50,0 | 400 | 97,9 |
|  | gidA-2 | GAACGTGGTTTCCCAATCAG |  | 60,4 | 50,0 | 400 |  |
| *radA* | radA-1 | GTCGCCGCCTAATAGGGTAA | 108 | 61,3 | 55,0 | 500 | 105,6 |
|  | radA-2 | ACCATAGAGCTGCGAGAGGA |  | 60,1 | 55,0 | 500 |  |
| *map* | map-1 | AAACGCGTCTGTCAAGCATC | 156 | 61,4 | 50,0 | 200 | 92,2 |
|  | map-2 | ACCCACACCGTGACCTACAA |  | 61,3 | 55,0 | 200 |  |
